# A Review on Risk Factors, Traditional Diagnostic Techniques, and Biomarkers for Pneumonia Prognostication and Management in Diabetic Patients

**DOI:** 10.3390/diseases12120310

**Published:** 2024-12-02

**Authors:** Shehwaz Anwar, Fahad A. Alhumaydhi, Arshad Husain Rahmani, Vikalp Kumar, Faris Alrumaihi

**Affiliations:** 1 Department of Medical Laboratory Technology, College of Nursing and Paramedical Sciences, Bareilly 243302, Uttar Pradesh, India; 2 Department of Medical Laboratories, College of Applied Medical Sciences, Qassim University, Buraydah 51452, Saudi Arabia; f.alhumaydhi@qu.edu.sa (F.A.A.); ah.rahmani@qu.edu.sa (A.H.R.)

**Keywords:** diabetes mellitus, diabetes-induced pneumonia, risk factors, traditional diagnostic techniques, biomarkers

## Abstract

People of all ages can contract pneumonia, and it can cause mild to severe disease and even death. In addition to being a major cause of death for elderly people and those with prior medical conditions such as diabetes, it isthe world’s biggest infectious cause of death for children. Diabetes mellitus is a metabolic condition with a high glucose level and is a leading cause of lower limb amputation, heart attacks, strokes, blindness, and renal failure. Hyperglycemia is known to impair neutrophil activity, damage antioxidant status, and weaken the humoral immune system. Therefore, diabetic patients are more susceptible to pneumonia than people without diabetes and linked fatalities. The absence of quick, precise, simple, and affordable ways to identify the etiologic agents of community-acquired pneumonia has made diagnostic studies’ usefulness contentious. Improvements in biological markers and molecular testing techniques have significantly increased the ability to diagnose pneumonia and other related respiratory infections. Identifying the risk factors for developing severe pneumonia and early testing in diabetic patients might lead to a significant decrease in the mortality of diabetic patients with pneumonia. In this regard, various risk factors, traditional testing techniques, and pathomechanisms are discussed in this review. Further, biomarkers and next-generation sequencing are briefly summarized. Finding biomarkers with the ability to distinguish between bacterial and viral pneumonia could be crucial because identifying the precise pathogen would stop the unnecessary use of antibiotics and effectively save the patient’s life.

## 1. Introduction

The incidence of diabetes mellitus (DM) has increased dramatically, making it a serious global public health concern [1]. Elevated blood glucose levels are an indication of DM, which over time damages the heart, blood vessels, eyes, kidneys, neurons, and other organs [2]. The organs and tissues impacted by type 2 diabetes mellitus (T2DM) include the brain, small intestine, kidneys, liver, skeletal muscle, adipose tissue, and pancreas [3]. More studies show that changes in gut flora, cytokine dysregulation, and inflammation are significant pathogenic contributors, especially in diabetics. Key indicators of diabetes include hyperglycemia, varied levels of insulin resistance, and decreased insulin production [2]. Hyperglycemia has been known to be one of the main causes of decreased immunity of diabetic patients. As a result, diabetes makes them more susceptible to infections such as pneumonia and pulmonary tuberculosis [4]. The innate immune system is frequently regarded as its first line of defense against prospective diseases and viruses [5]. On the other hand, it is also necessary for the later establishment of the adaptive response to pathogens encountered, which is accomplished by the proliferation of particular B- and T-lymphocyte clones. The innate immune response functions by means of a phagocytic cell population, a set of proteins, and a mechanism that can identify conserved characteristics of infections. This allows the immune system to take immediate action against pathogens upon encounter with the host [6]. People with diabetes have impaired immune systems. People with uncontrolled diabetes become immunocompromised due to the detrimental impacts of a high blood sugar level environment which induces immunological dysfunction, like decreased neutrophil activity, an impaired antioxidant system, and a weakened humoral immune system [7,8]. Therefore, patients with DM are more susceptible to various infections such as respiratory infections, malignant external otitis, rhino-cerebral mucormycosis, or emphysematous infections of the gallbladder, kidney, urinary bladder, renal disease, vascular disease, and lower extremity amputations [9]. Several immune system functions, including polymorphonuclear leukocyte function (including chemotaxis, phagocytosis, and leukocyte adhesion) and bactericidal activity in the serum, have been shown to be impaired [10].

Pneumonia is the most severe type of acute lower respiratory tract infection, affecting the pulmonary parenchyma in lungs. It is a widespread disease with a high risk of infection and significant morbidity and mortality. Pneumonia is the sixth leading cause of death [11]. Fever, coughing, and purulent fluid infiltration of the alveoli due to microbial pathogen infection are the clinical manifestations of pneumonia [12] and pneumonia may attack one or both lungs. Pneumonia that affects both lungs can be referred to clinically as bilateral or double pneumonia. Nasal carriers, sinusitis, oropharynx, stomach or tracheal colonization, and hematogenous spread have been considered to be a few examples of endogenous sources of pathogens [13]. When pathogens penetrate the lower respiratory tract and lung parenchyma at the alveolar level, the body responds by initiating an inflammatory attack. Further, a number of host defenses work together in the lungs to stop infections from spreading. Alveolar macrophages, immune cells that neutralize and eliminate bacterial growth, are the primary component of the pulmonary defense system. However, infections spread as soon as they overcome the body’s defenses [14].

The inflammatory response induced by alveolar macrophages in this situation is known to improve the lower respiratory tract defenses. This inflammatory response is the primary factor in the clinical presentation of bacterial pneumonia. Interleukin-1 (IL-1) and tumor necrosis factor (TNF) are two molecules that may trigger fever. The release of cytokines in response to the inflammatory response causes physiological symptoms. IL-8 (interleukin-8) and colony-stimulating factors such as G-CSF (granulocyte colony-stimulating factor) stimulate neutrophil chemotaxis and maturation, resulting in leukocytosis in serological tests and purulent exudates [15]. The presence of these cytokines causes shortness of breath by promoting leaking in the alveolar–capillary membrane at the site of inflammation [16].

It is well documented that there is a link between infections and diabetes, with infections accounting for a significant factor in diabetes-related death and sickness. Almost all infections in diabetics result in substantial mortality rates if not detected and treated appropriately. Conversely, diabetes frequently worsens the severity of infections [6]. The effectiveness of vaccines and factors affecting their efficacy in diabetics are unknown. Additionally, it is unclear whether the immune response of diabetic patients is weakened to these vaccines. Pneumonia is more likely to strike adults with chronic medical conditions like diabetes [14].

In the general population, DM is a powerful risk factor for pneumonia-related deaths in the elderly. To further understand the underlying mechanisms causing the higher mortality associated with pneumonia in people with type 2 diabetes, more research is required [10]. Finding patients with risk factors for multidrug-resistant organisms is essential to assuring accurate, effective treatment. Uncertainty in diagnosis, particularly in relation to VAP, probably contributes to antibiotic overuse and increases the risk of antibiotic-associated damage. Rapid diagnostic techniques offer the potential for more focused, particular treatment and fewer needless medications [17]. A coordinated focus and investment in acute respiratory infection research are now required since the inadequate evidence foundation supporting the majority of therapeutic decisions in these conditions can no longer be justified [18].

Successful pharmacologic selection and treatment of pneumonia depend on an understanding of the local bacterial pathogens, antibiotic sensitivity, and resistance profiles [19]. The consequences of novel vaccines, medical treatments, and diagnostics will be severely limited if mortality and access to care are negatively related. More knowledge of this, together with more specific details regarding the pathophysiology and etiology of the disease, should direct innovative strategies to address the massive worldwide issue of pneumonia-related fatalities [20]. Improved knowledge of the ever-growing lists of host susceptibility factors, the effects of individual host responses to pneumonia, and the long-term medical consequences of lung infections are all necessary to reduce the worldwide burden of pneumonia. An integrated approach that addresses both the host’s reactions to pneumonia and the etiological pathogen of pneumonia is necessary to advance our future therapy of the disease [21]. Therefore, the current review aims to summarize various causes and risk factors, as well as traditional diagnostic strategies. Additionally, a brief discussion of biomarkers and next-generation sequencing used for diagnosis of pneumonia is provided at the end of this review.

**Scheme 1:**
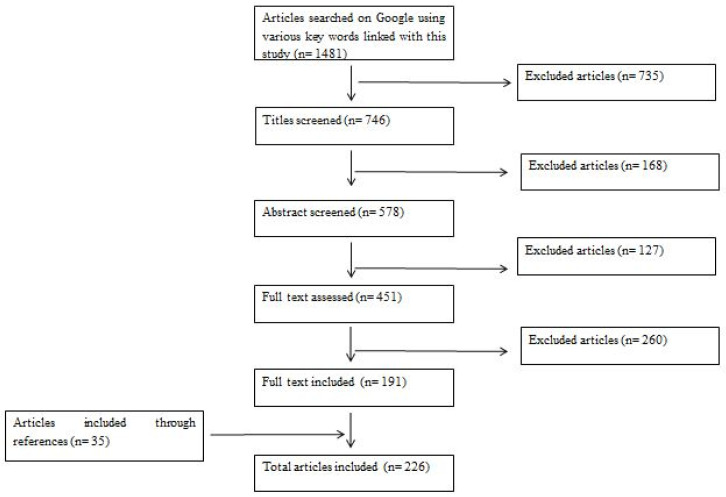
A summary of inclusion methods for articles.

This work is a general review article, and it is a written synthesis and assessment of published works on risk factors, etiology, traditional diagnostic techniques, and biomarkers for pneumonia prognostication and management in diabetic patients. It discusses, summarizes, and evaluates earlier studies, research, theories, and hypotheses linked to this topic. In addition, it identifies contentious issues and assertions. It gives a summary of what is currently known, which can be helpful to find pertinent theories, approaches, and research gaps. During the writing of this manuscript, the Google search engine was used, and the main focus and priority were given to research and studies brought on after 2016. The keywords included were diabetes, causes of pneumonia in the diabetes group, pathomechanism of pneumonia, types of pneumonia, risk factors for pneumonia infection in the diabetic group, clinical predictors, signs and symptoms of pneumonia infection, and strategies to diagnose pneumonia infection. Further, biomarkers and next-generation sequencing for detection and identification of therapeutic targets were used. Scheme 1 gives a summary of methods for including articles in this manuscript.

## 2. Causes of Pneumonia

**Figure 1:**
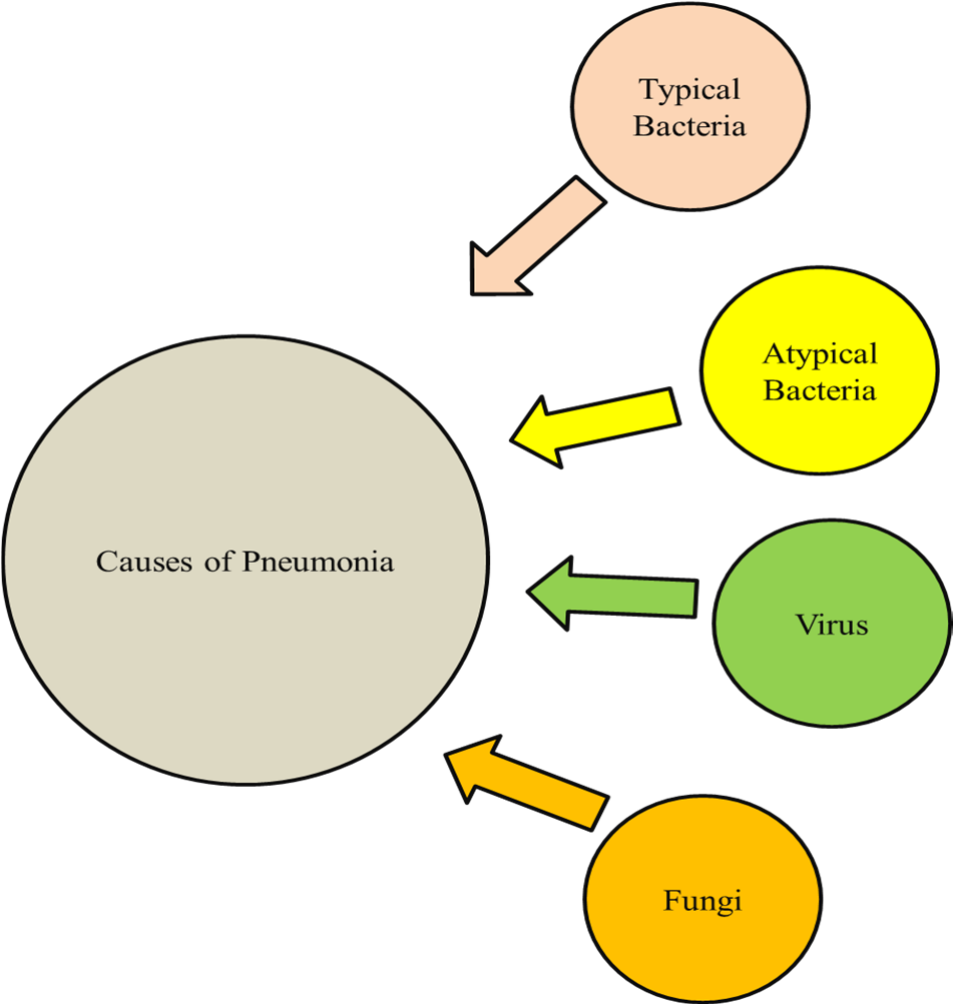
Causes of pneumonia in diabetic patients.

Numerous infectious organisms, such as bacteria, fungi, and viruses, can cause pneumonia (Figure 1). The most prevalent pathogens are *Streptococcus pneumonia* (pneumococcus) and respiratory viruses. Patients with community-acquired pneumonia (CAP) are the most common hosts of these pathogens. Nevertheless, despite detailed microbiologic research, a sizable percentage of patients (up to 62% in certain studies) were determined to be pathogen-free. In general, the most often identified causes of CAP can be divided into three groups [22]. Here, parasitic pneumonia is additionally discussed, which is not included in Figure 1.

### 2.1. Typical Bacteria

*Streptococcus pneumoniae* is the most common bacterial cause of bacterial pneumonia. Other bacterial pathogens that cause the disease include *Haemophilus influenzae*, *Moraxella catarrhalis*, *Staphylococcus aureus*, aerobic gram-negative bacteria, microaerophilic bacteria, and anaerobic Enterobacteriaceae like *Klebsiella* spp. and *Escherichia coli* [23].

### 2.2. Atypical Bacteria

They are categorized as “unusual” due to their innate resistance to beta-lactams and the fact that they cannot be detected using Gram staining or conventional cultivation techniques. *Chlamydia psittaci*, *Mycoplasma pneumoniae*, *Legionella* species, and *Coxiella burnetii* are a few examples [24].

### 2.3. Respiratory Viruses

Adenoviruses, influenza A and B viruses, SARS-CoV-2, and other coronaviruses (specifically CoV-229E, NL63, OC43, and HKU1), as well as rhinoviruses, parainfluenza viruses, human bocaviruses, human metapneumovirus, and respiratory syncytial virus, have all been linked to a significant prevalence of respiratory diseases in patients with pneumonitis, according to ongoing observation of these patients [25].

### 2.4. Mycotic Causes

Pneumocystis, Cryptococcus, and Aspergillus are the three most frequent fungi that cause fungal pneumonia. These fungi can be found in soil, the air, and medical settings like hospitals (https://www.verywellhealth.com/fungal-pneumonia-5179190#:~:text=Several%20fungi%20cause%20fungal%20pneumonia,and%20clinical%20environments%20like%20hospitals, accessed on 19 September 2024). *Pneumocystis jiroveci* is one of the most typical causes of pneumonia in HIV-infected newborns, accounting for at least 25% of all pneumonia-related deaths. Geographical location, pneumococcal vaccination rates, host risk factors (such as smoking), season, and pneumonia severity all affect the relative incidence of these infections.

### 2.5. Parasitic Cause: Parasitic Pneumonia

Multiple types of parasites are known to be implicated in lung diseases, and these kinds of lung diseases caused by parasitic infections are known as parasitic lung diseases. The presence of parasites in the lungs or weakened immunity brought on by parasitic infections could be the most likely reason for such diseases. Parasitic lung infections are very common in impoverished nations due to various parasites found in their environment. However, parasitic lung patients might be noticed in industrialized nations due to increased immigration, globalization, worldwide travel, etc. Radiographic abnormalities are the most prevalent symptoms, though isolated pulmonary nodules may also occur [26]. With a few exceptions, protozoal and helminthic pneumonia are more common in tropical countries and are known to affect more immune-compromised patients [27]. Lung parasite infections can impact the respiratory system, and their clinical manifestations may resemble cancer and tuberculosis. Since the majority of instances of parasite pneumonia are treatable with medical intervention, it is crucial to detect the disease early [28].

The common parasitic infections affecting the lung have been reported to be caused by protozoa, nematodes, and trematodes [29]. In particular, parasites such as Toxocora, Trypnosoma, amoeba, Schistosoma, Leishmania, and malaria could lead to parasitic lung diseases. Additionally, an infection of Ancylostoma or Toxocora can be contracted by exposure to cats and dogs. Churg–Strauss syndrome could be involved in multiple organ damage [27].

#### 2.5.1. Protozoal Pneumonia

Protozoa can affect the respiratory system, and this kind of respiratory disease is very commonly noticed in cases of patients having conditions with immunosuppression, such as AIDS, organ transplant, malignant hemopathies, corticotherapy, etc. In addition, immigration and travel to endemic regions are another factor that can lead to protozoal respiratory diseases. Protozoa can affect the respiratory system through various mechanisms. They can either directly damage parenchyma or can raise systemic inflammatory response through hematogenous spread. Further, by being next to a lesion (like amoebiasis), protozoa can also have an impact on the respiratory system [30]. There is extensive documentation of pulmonary infections caused by free-living amoebas, Toxoplasma species, Babesia species, Cryptosporidium species, Leishmania species, and Microsporidia species [31].

#### 2.5.2. Helminths in Respiratory Diseases

Helminth infections continue to be a major worldwide problem that affects billions of people’s lives. Pulmonary diseases are among the severe morbidities caused by a number of human helminth infections. Helminth-induced lung pathology might be localized lung lesions, widespread lung disease, or hypersensitivity reactions and systemic inflammation. Lung disease-causing helminths usually migrate as larvae either directly through the lung parenchyma or through the lung vasculature, develop cysts and nodules in the lung tissue, or affect the lungs indirectly [32].

Numerous investigations on felid lungworms have been carried out in recent years, and the results have yielded significant and new information for clinical settings. At the same time, fresh knowledge has led to the introduction of new viewpoints, and further research is required soon in order to apply diagnostic and control approaches [33]. Cardio-pulmonary nematodes such as *Eucoleus aerophilus*, *Crenosoma vulpis*, and *Angiostrongylus vasorum* (*A. vasorum*) are common parasites in household dogs. Mild respiratory symptoms like coughing, sneezing, dyspnea, etc., can be followed by more severe ones like pulmonary hypertension in dogs infected with *A. vasorum* [34]. Tropical pulmonary eosinophilia (TPE) is a hyperrespiratory condition, and it is a hidden sign of lymphatic filariasis (LF) that is brought on by *Wuchereria bancrofti* (*W. bancrofti*) and *Brugia malayi*. Research indicates that less than 0.5 percent of the 130 million filariasis patients worldwide appear to acquire TPE [35]. A new compound from *W. bancrofti* microfilariae was discovered by Katru and associates, and it was found to be bonded to monoclonal IgE antibodies made from human filaria-infected individuals [36]. *W. bancrofti* microfilariae were remarkably visible in the centrifuged fluid debris microscopy of a 46-year-old male in India. The man was suffering from malaise, fever, chest pain, shortness of breath, and generalized weakness for six months and had a past history of pulmonary tuberculosis and DM. His respiratory symptoms and effusion were reported to be totally resolved after taking antihelminthic medicine for six to eight months [37].

Trematodes are a broad category of parasites that have an impact on the health of people and animals all over the world. According to one theory, trematodes developed from free-living flatworms, which are the ancestors of modern *rhabdocoel turbellarians*, and then became closely related to mollusks until they eventually evolved into parasitic organisms. The colon, lungs, liver, and vascular system are among the many organs in the vertebrate, the final host, where these worms are located. These parasite infections cause significant losses in livestock industry productivity, as well as a decline in human quality of life [38]. Greater Caribbean manatees with severe nasal *Pulmonicola cochleotrema* infections and unusual respiratory sounds were clinically suspected of having pneumonia in Brazil [39].

## 3. Risk Factors of Pneumonia Infection

**Figure 2:**
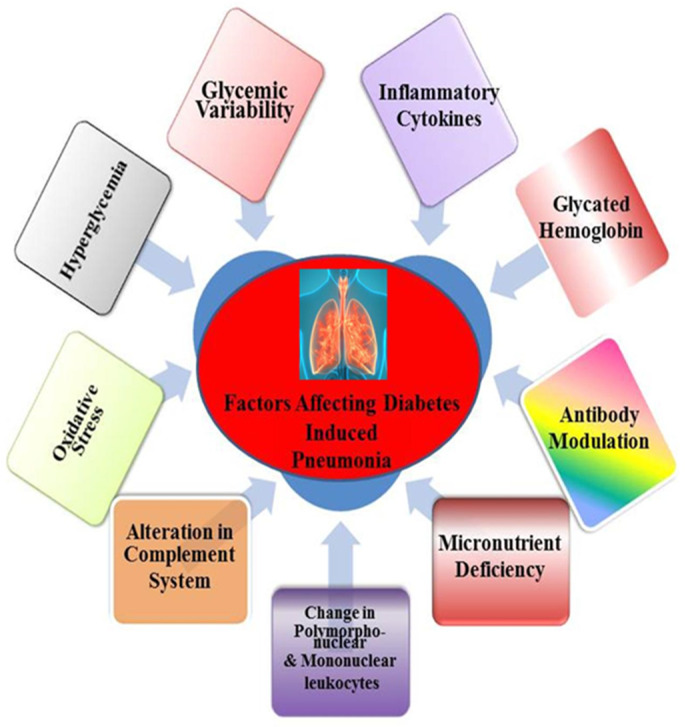
Risk factors for acquiring pneumonia and its severity in diabetic patients.

Although there are limited and conflicting data available, diabetes is regarded to be an important risk factor for pneumonia. Pneumonia may be more common in diabetic patients due to several reasons. Aspiration, hyperglycemia, lowered immunity, compromised lung function, pulmonary microangiopathy, and concurrent morbidity are among the risks faced by diabetic patients. Numerous risk factors contribute to the severity of infections in diabetic patients (Figure 2). Critical risk factors are summarized in Table 1.

### 3.1. Hyperglycemia

Hyperglycemia has been reported to be linked to high fatality rate and complications in individuals with pneumonia [49]. By causing systemic inflammation, hyperglycemia impairs pulmonary function [50]. Hyperglycemia has been implicated in the glycation process that results in acute respiratory distress syndrome and patient mortality in patients with COVID-19 [51]. Further, pulmonary dysfunction and structural changes in lung tissue like collapse of portions of the lung and augmented permeability of the blood vessels are induced by hyperglycemia. Hyperglycemia leads to an increase in TNF- and IL-6, which results in impaired immune system function. These changes may ultimately result in an elevated risk of infection, slowed wound healing, multiple organ failure, an extended hospital stay, and even death [52]. In patients, hyperglycemia is typical and has been linked to higher rates of morbidity and death in both diabetic and non-diabetic patients [53].

Pneumonia has previously been linked to diabetes mellitus (DM) as a risk factor. According to several studies, CAP increases the mortality rate of patients, and it has also been linked to poor CAP outcomes in patients who had hyperglycemia at the time of admission [54]. Hyperglycemia is a common complication in community-acquired pneumonia (CAP) patients, which can lead to serious outcomes. A greater probability of ICU admission and a propensity for a connection with in-hospital mortality were both linked to hyperglycemia in non-diabetic patients. Stress-induced hyperglycemia can be brought on by a rise in catecholamine, glucagon, growth hormone, pro-inflammatory cytokines, and peripheral insulin resistance in response to physiological stress. Furthermore, pneumonia may also be caused by systemic bacterial and viral infections, which are strong stress hyperglycemia triggers [40]. In patients admitted with both non-COVID-19 CAP and in-hospital hyperglycemia, there is a higher risk of in-hospital morbidity and death as compared with patients having diabetes alone. Diabetes also weakens the innate and humoral immune systems by reducing the activity of neutrophils and T-lymphocytes. The primary functions of the innate immune system, including phagocytosis, chemotaxis, and the bactericidal capacities of neutrophils and macrophages, are impaired by hyperglycemia, which leads to secondary bacterial infection. Bacterial infections may become more severe and common as a result of hyperglycemia [55].

The level of glucose in airway secretions can be immediately elevated by hyperglycemia. Hyperglycemia may act as a viral replication booster because it has been discovered that high glucose levels dramatically accelerate influenza virus infection and proliferation in pulmonary epithelial cells. Hypoglycemia promotes pathogen growth in the lungs, increases virus replication, and raises the likelihood of subsequent bacterial infection [56].

### 3.2. Glycemic Variability

Mendez and colleagues looked into the relationship between glycemic variability and non-critically ill hospitalized patients’ length of stay and 90-day mortality. According to the findings, individuals who are not critically ill have longer hospital stays and a higher mortality rate when their GV is higher [53]. The impact of glycemic variability (GV) on hospital mortality and duration of stay for non-critical diabetic patients was examined in a study. The results of diabetes patients admitted with community-acquired pneumonia or an acute aggravation of chronic obstructive lung disease were confirmed by this investigation to be negatively impacted by glycemic fluctuation [57].

A prolonged stay in the intensive care unit and higher mortality risk were linked to significant glycemic variability within 48 h of ICU admission. However, in critically ill patients with pneumonia, early-phase hyperglycemia variations may be minimized [41]. A high GV is positively associated with pneumonia patients being in the hospital for a longer period of time. Patients with CAP who are receiving glucocorticoids, particularly those with type 2 diabetic mellitus (T2DM), are at an increased risk of experiencing high GV and need to receive medical treatment to reduce GV. This is especially true during the day [58].

### 3.3. Oxidative Stress

The term “oxidative stress” describes the excessive generation and depletion of free radicals, such as reactive oxygen species (ROS), brought on by a malfunctioning antioxidant defense mechanism. Free radicals and ROS are frequently produced by regular cellular metabolism and are essential for a number of cell signaling pathways [59,60]. The free radical theory of aging postulates that ROS are produced during various oxidative reactions in living organisms [61,62].

Proteins, lipids, carbohydrates, and DNA may all be affected by oxidative stress, and oxidative stress is defined as an imbalance between antioxidants and oxidants [63]. The location, structure, and function of the lung make it a more vulnerable organ to oxidative injury. Conversely, diabetes increases the risk of viral pneumonia and causes inflammation and oxidative stress in the lungs. In addition, hyperglycemia can cause a number of pulmonary problems, such as decreased lung aspiratory performance, increased pulmonary microangiopathy, damaged lung structure, impaired immunity, and increased levels of oxidative stress [64]. Superoxide dismutases are important antioxidants that are working as chief antioxidant defenses against oxidative stress [65]. Patients with pneumonia have been experienced to have increased inflammation and oxidative stress [66].

The primary pathogenic mechanism underlying the development and progression of pneumonia includes diabetes-induced oxidative stress, changes in redox signaling, and inflammatory reactions [67]. Redox-sensitive mediators are altered by oxidative stress in the airways, which leads to lung dysfunction. Furthermore, hyperglycemia causes oxidative stress by increasing the concentration of mitochondrial superoxide anion, increasing protein glycation, and activating a number of signaling pathways that may alter pulmonary function and structure [50]. Apoptosis of alveolar epithelial cells can also be brought on by elevated mitochondrial oxidative stress. The alveolar membrane gets damaged by the death of epithelial cells. By increasing the amount of extracellular matrix, the fibroblasts help to repair this damage. The fibroblasts’ resistance to apoptosis may be the cause of the matrix’s persistent deposition, which damages the lung’s healthy structure [68].

### 3.4. Glycation

Glycation plays an important role in various oxidative stress-related diseases [69,70]. T2DM-induced glycated serum hemoglobin impairs immunity and increases susceptibility to bacterial infections, such as *Streptococcus pneumoniae*. The investigation looked at how people who had or did not have diabetes responded to CD4+ and T-helper 17 cells. The percentage of glycated hemoglobin A1c (HbA1c) and increased glucose levels are associated with reduced CD4+ and T-helper 17 cell memory response to *Streptococcus pneumoniae* in Mexican Americans with type 2 diabetes [71].

Additionally, hyperglycemia stimulates the production of collagen by inducing oxidative stress (OS), increasing NF-kB gene expression, and enhancing inflammatory factors. It also encourages the formation of cross-links by accelerating the production of AGEs, which ultimately impairs lung function [50]. In order to identify the relationship between HbA1c levels and exacerbation status in patients with chronic obstructive pulmonary disease (COPD), Motamed and colleagues conducted a study on a group of COPD patients. The results show that the percentage of HbA1c was linked to COPD exacerbations and that HbA1c was a reliable indicator of the severity of the disease in COPD patients [72]. In British individuals with asthma, glycated hemoglobin (HbA1c) is associated with asthma-related hospitalizations and minor declines in FEV1 and FVC [73]. In COVID-19 patients, HbA1c acts as a predictor of mortality in patients with severe pneumonia [74]. Additionally, COVID-19 confirmed that HbA1c is a predictor of mortality among individuals with severe pneumonia [75]. A study was conducted in 2021 to determine the impact of HbA1c and the length of diabetes on lung function in T2DM patients, as well as to determine whether long-term diabetes or high HbA1c levels are more harmful to the lung functions. Lung function and DM were reported to be related. The results show that compared to the duration of DM, high HbA1c or uncontrolled DM had a greater detrimental influence on pulmonary function impairment [76].

### 3.5. Abnormal Complement

One of the primary processes producing humoral resistance is the complement system. It is made up of serum and surface proteins whose main functions are to encourage macrophages and neutrophils to attack and phagocytoze bacteria and to induce bacterial lysis. Additionally, the second signal for B-lymphocyte activation and antibody production comes from complement activation products. Although some studies have found a shortage of the C4 component in DM, this loss of C4 is likely linked to lower cytokine response and polymorphonuclear dysfunction [77]. Immunity, including complement activation, has become more widely recognized in conditions such as ischemia reperfusion injury (IRI), acute lung injury (ALI), acute respiratory distress syndrome (ARDS), pneumonia, asthma, pulmonary arterial hypertension (PAH), idiopathic pulmonary fibrosis (IPF), chronic obstructive pulmonary disease (COPD), and obliterative bronchiolitis (OB) [78].

The complement cascade is known to be activated by three pathways. The C1q protein binds to the Fc receptor on the antibody–antigen complex to start the classical route. Mannose-binding lectin, which binds to mannose (carbohydrates) on pathogens’ surface, activates the lectin pathway. The alternative pathway uses spontaneous C3 hydrolysis as an amplification loop to start the complement activation process. As a result of the convergence of all three pathways, C3 convertase is formed, which later results in the assembly of C5 convertase. This results in the formation of the membrane attack complex (MAC), which causes cell death [79]. The immune system is seriously affected by hyperglycemia because it collaborates in vitro with components of the innate immune system as well as the adaptive immune system, thereby inhibiting the functions of T-lymphocytes, immunoglobulin, and complements [80].

Immunoglobulin and the complement system, commonly referred to as hemoral immunity, play an essential role for both avoidance and management of infections of the respiratory tract. Infections of lung tissue brought on by encapsulated bacteria are more probable to occur in people with deficiencies in humoral immunity, such as common variable immunodeficiency [81]. Current vaccines typically prevent pneumonia by stimulating the development of antibodies. C3 and C5 convertase, as well as MAC, can be used to control the complement pathways [82].

Numerous viruses, bacteria, and fungi have biochemical characteristics that can activate complement proteins and trigger the innate immune response to defend the host. A total of 39 people who had pneumococcal pneumonia had their levels of complement proteins and the alternate complement pathway’s functional activity evaluated. Properdin and factor B mean values were considerably lower in the 19 patients with pneumococcal pneumonia and bacteremia than they were in the 20 patients without bacteremia, indicating a more severe depression of the alternative complement pathway in the presence of bacteremia. These findings support the idea that the alternative route plays a significant role in host defenses during pneumococcal infection by showing a selective suppression of the pathway in patients with pneumococcal pneumonia. Low serum levels of properdin and factor B, which are implicated in the alternative route of the complement system in pneumococcal pneumonia, are linked to bacteremia [83]. Highly pure pneumolysin (at a concentration of 10 micrograms/mL) significantly activated the human complement system, as determined by the conversion of C3 [84].

### 3.6. Abnormal Inflammatory Cytokines

Complex interactions between immune cells and both pro-inflammatory and anti-inflammatory cytokines are essential for the regulation of the inflammation that occurs in bacterial pneumonia. Tumor necrosis factor α (TNFα) and interleukin 1 (IL-1), two crucial pro-inflammatory cytokines and early response mediators, control numerous cellular processes and direct actions resulting in the beginning, maintenance, and repair of tissue injury. A competitive occupancy of the IL-1 receptor without agonist action is caused by the interleukin 1 receptor antagonist (IL-1ra). A rise in anti-inflammatory cytokines in CAP may be attributed to PMN. Strategies to boost neutrophil numbers may have positive benefits by altering the inflammatory cytokine response, as well as enhancing the antibacterial activity [85].

Surface protein A and surface protein D double knockout (SP-A/D KO) mice were used in Du and colleagues’ study, which involved either *S. aureus* or a dummy strain of the disease. In SP-A/D KO mice with pneumonia, compared to WT controls, there was an increase in the expression and nuclear translocation of nuclear factor kB (NF-kB), p65, the gut levels of tumor necrosis factor a, and interleukin-1b [86]. When triggered by lipo-polysaccharides, mononuclear cells and monocytes from people with diabetes release less interleukin-1 (IL-1) and IL-6. It appears that DM individuals’ cells have an inherent abnormality that causes them to produce less interleukins that than healthy cells do [77].

### 3.7. Abnormal Mono- and Polymorphonuclear Leukocytes

Hyperglycemia may result in decreased chemotaxis, phagocytic activity, and polymorphonuclear leukocyte mobilization. By suppressing glucose-6-phosphate dehydrogenase (G6PD), increasing polymorphonuclear leukocyte death, and diminishing polymorphonuclear leukocyte transmigration through the endothelium, the hyperglycemic environment also prevents the antibacterial function from functioning [6]. The hyperglycemic environment elevates intracellular glucose levels, which are subsequently processed using NADPH as a cofactor in tissues that do not require insulin for glucose transport. The susceptibility of cells to oxidative stress increases as NADPH levels fall because this prevents the regeneration of molecules which are essential to the cell’s protective antioxidant mechanisms [77].

Polymorphonuclear leukocytes (PMNs) accumulate in the lung and migrate into the alveolar airspaces during acute bacterial pneumonia. However, PMNs may take a while to move into the airspaces at the inflammatory site after being released into the circulation as part of the systemic response to a local streptococcal pneumonia [87]. Using 5’-bromo-2’-deoxyuridine (BrdU), a study was carried out to gauge the transit time of PMNs in the mitotic and post-mitotic pools of the BM in rabbits. The release of immature PMNs with greater levels of lysosomal enzymes into the circulation is possible as a result of *S. pneumoniae* reducing the transit time of PMNs in the mitotic and post-mitotic pools in the bone marrow [88].

Particularly in individuals with immune-compromised conditions like diabetic patients, *S. aureus* can cause peritonitis and mortality. Male Wistar rats with alloxan diabetes and the corresponding controls were injected intraperitoneally with several *S. aureus* strains or sterile phosphate-buffered saline solutions. The levels of IL-1, IL-6, and IFN- were raised in the PeLF of diabetic rats following *S. aureus* infection after several insulin treatments. In the course of peritonitis brought on by several strains of *S. aureus*, PMN leukocyte movement and adhesion molecule expression have been identified [89].

### 3.8. Antibody Impairment or Reduced Antibody Response

In proportion to an increase in HbA1c, immunoglobulin glycation occurs in diabetic individuals, which could be harmful to the biological function of the antibodies. However, it is uncertain how clinically important these findings are, given that individuals with diabetes have suitable antibody responses to vaccinations and frequent illnesses [77]. A study investigated the baseline protective levels of antibodies against both the capsular polysaccharide and the *S. pneumoniae* surface protein A (PspA) in people with and without type 2 diabetes mellitus. In Mexican American adults with T2DM, impaired function of antibodies to pneumococcal surface protein A but not to capsular polysaccharide was observed. The findings indicate a connection between diabetes and a reduced antibody response [90].

Since a lengthy time ago, meningitis and bacteremia in children have been mostly caused by the encapsulated bacteria *S. pneumoniae*, *Neisseria meningitis*, *Haemophilus influenza*, and *Streptococcus agalactiae* (Group B Streptococcus). The exact mechanisms and contributions of these and other components vary between species. These mechanisms may include opsonophagocytosis and complement-dependent bacteriolysis. The protection against these bacteria is largely mediated by polysaccharide-specific antibodies and complements [91].

To provide broader, cross-serotype protection, a trivalent pneumococcal protein vaccine consisting of pneumococcal cholinebinding protein A (PcpA), pneumococcal histamine triad protein D (PhtD), and detoxified pneumolysin is being developed. Antibodies that neutralize *S. pneumoniae* (Spn) produce pneumolysin and protect against bacterial pneumonia. Antibodies against PhtD and anti-PcpA produced by pneumococcal protein vaccines additionally safeguard against Spn via a complement- and macrophage-dependent opsonophagocytosis [92]. Antibodies to protein antigens, rather than the capsule, are primarily accountable for naturally acquired protection against IPD (invasive pneumococcal disease) [93].

### 3.9. Deficiency of Micronutrient

Every stage of the immune response is also reliant on the availability or balance of micronutrients. In order to enable immunological effector actions, micronutrients work in concert with the molecular mechanism [94]. One of the most important micronutrients impacting the efficient functioning of immune responses is vitamin D, which has been receiving more attention. The heart, brain, pancreatic islets, immunological cells, muscles, and adipose tissue all have vitamin D receptors (VDRs), which control the inhibitory impact of the active form of vitamin D, calcitriol (1,25 (OH)2D3). In the onset of diabetes and its associated complications, they are all common target organs [95]. Normally, immune cells would activate TLR signaling during an infection to cause the generation of antimicrobial proteins like cathelicidin (IL-37) and VDR signaling to convert vitamin D into calcitriol [96].

As vitamin D insufficiency is linked to β-cell dysfunction and insulin resistance, several studies have found an inverse connection between vitamin D status and the prevalence of T2D. According to the outcomes of an observational study, there were a greater number of instances of hypovitaminosis D in T2DM patients than in controls (39% vs. 25%) [97]. To investigate potential factors, sensitivity analyses and a random-effect or fixed-effect meta-analysis were performed. There were eight observational studies with 20,966 participants total. In this meta-analysis, CAP patients with vitamin D deficiency had a significantly greater probability to acquire the medical condition, and CAP patients’ serum vitamin D levels definitely dropped by 5.63 ng/mL (95% CI: 9.11, 2.14). According to the findings from this meta-analysis, CAP patients are more likely to be suffering from insufficient levels of vitamin D [98].

Growing elderly and simultaneous conditions like heart failure, diabetes, neoplasia, and COPD are risk factors for developing community-acquired pneumonia. It has been discovered that zinc is crucial in the control of T-cell-mediated function. The immune system, protein synthesis, wound healing, DNA synthesis, and cell division are all influenced by zinc. In CAP, morbidity and mortality are correlated with zinc deficiency. Low serum levels are linked to pneumonia severity and related morbidity and mortality [99].

In contrast, Zhou et al. and Lassi et al. identified that high Zn levels in pediatric patients with pneumonia could reduce the incidence and prevalence of pneumonia, decrease the number of days spent in the hospital, and improve the clinical outcome [98,100]. When compared to healthy controls, critically ill people who have respiratory infections have been shown to have lower serum selenium levels. In addition, decreased blood selenium levels were significantly correlated with lower albumin levels and a decline in lymphocytes [67].

### 3.10. Metformin Use

Metformin is a vital metabolic regulator and has long been regarded as the first-line treatment for T2DM. Metformin activates adenosine 5’-monophosphate-activated protein kinase (AMPK), which in turn stimulates neutrophils and controls the release of cytokines that have antibacterial and anti-inflammatory properties [101]. Metformin pretreatment in diabetic mice may alter glucose flux across the airway epithelium and reduce the development of bacteria brought on by hyperglycemia [102].

In 2022, the use of metformin was found to be linked to considerably lower chances of bacterial pneumonia, invasive mechanical ventilation, and respiratory cause of death in individuals with T2DM [103]. Patients with T2DM who use metformin have a dose–response pattern of decreased risk of tuberculosis infection [104].

### 3.11. Neuropathy

As a result of decreased physiological mechanisms leading to an overexpressed response to inflammatory stimuli, diabetic neuropathy may be more significant in the loss of immune response regulation capacity. Thickness of the alveolar epithelium and pulmonary capillary basal laminae is one of the most commonly reported histological pulmonary abnormalities in diabetes, and it is correlated with the degree of microvascular problems. Functional changes in the control of breathing and pulmonary bronchomotor tone may be brought on by diabetic autonomic neuropathy [105]. An analysis of patients with T2DM and concurrent CAP revealed that advanced age, altered consciousness, elevated pulse rate, acidosis, elevated neutrophil–lymphocyte ratio, hyponatremia, hyperglycemia, and diabetic nephropathy were among the risk factors for in-hospital death [106].

## 4. Clinical Predictors of Pneumonia

In poor nations, research has been conducted on the sensitivity and specificity of pneumonia, as well as its clinical symptoms and indications. The clinical signs and symptoms of CAP can range greatly, from mild pneumonia with symptoms like fever, coughing, and shortness of breath to severe pneumonia with signs and symptoms including sepsis and respiratory distress (Figure 2). The degree of the patient’s individual local and systemic immune response has a strong association with the severity of their symptoms. The most typical sign of pneumonia is a cough that produces green, yellow, or bloody mucus [107]. Fever, shivering chills, shortness of breath, low energy, and extreme fatigue are additional symptoms. Among the most common manifestations of CAP are cough (with or without sputum production), dyspnea, and pleuritic discomfort in the chest. Tachypnea, increased breathing effort, and erratic breath noises, such as rales, crackles, and rhonchi, are all symptoms of pneumonia. Additionally suggestive of pneumonia are tactile fremitus, egophony, and dullness to percussion. The buildup of white blood cells (WBCs), fluid, and proteins in the alveolar space is the cause of many symptoms and indications. The consequent impairment of alveolar gas exchange may lead to hypoxemia. Pulmonary opacities are chest radiographs that show the buildup of WBCs and fluid within the alveoli [108].

A history of coughing or shortness of breath, difficulty in swallowing, a high respiratory rate, drawing of the lower chest wall, a temperature, and tachycardia are documented to be some symptoms and indicators of pneumonia. According to Htun et al. (2019), adult radiographic pneumonia can only be identified by a concomitant set of the symptoms, such as cough, pyrexia, tachycardia, tachypnea, and crackles [16]. According to some reports, hypoxemia, nasal discharge, reduced breath sounds, noisy breathing, and groaning are all associated with radiographic pneumonia. However, hypoxemia is a reliable predictor after controlling for all other factors [109].

People with T2DM have been reported to show different CBC parameters as compared with non-diabetic people. Generally, people with T2D show higher WBC counts and lower RBC counts, hemoglobin, HCT, MCV, and MCH than healthy people. Further, RBC distribution width, HbA1C, and fasting blood sugar have been reported to be greater in T2DM patients as compared with the healthy population [110]. In one study, the sample consisted of 212 pneumonia patients without diabetes and 61 pneumonia patients with diabetes. It was discovered that sixty-six distinct bacteria were linked to severe pneumonia in diabetic patients [111]. In 2011, a report regarding a comparative study of bacterial pneumonia in 50 patients with diabetes and 50 patients without diabetes was published. This report showed that pneumonia patients with diabetes had elevated BUN. Additionally, the diabetic group had a higher prevalence of multi-lobe involvement. Gram staining revealed that the non-diabetic group had considerably more gram-positive cocci than the diabetic group [112].

Using clinical, imaging, and laboratory features of individuals with confirmed COVID-19, a study was carried out to investigate if comorbidity with type 2 diabetes (T2D) influences the clinical and hematological parameters of COVID-19 patients. T2DM patients needed more time to recover from pneumonia and had more chances of subsequent bacterial infection. White blood cell and neutrophil counts were considerably greater, and there was more severe inflammation as well as lymphocytopenia in the T2DM group. Nonetheless, hyperlactatemia, hyponatremia, and hypocalcemia appeared to be considerably more common in T2D individuals [113].

A study examined the relationship between diabetes mellitus and COVID-19 infection. Fever, anorexia, dry cough, shortness of breath, and chest pain were more common in DM patients. When compared to non-diabetics, the mean of hematological and biochemical parameters, including hemoglobin, calcium, and alkaline phosphate, were reported to be significantly decreased in diabetics, while other parameters, including glucose, potassium, and cardiac troponin, significantly increased [114].

## 5. Systemic Symptoms and Signs

Chills, fever, exhaustion, malaise, anorexia, and systemic inflammatory response linked with tachycardia are among the other prevalent systemic symptoms. Other observations include leukopenia or leukocytosis with a leftward shift. In addition, procalcitonin is primarily associated with bacterial infections. Other inflammatory markers, such as the erythrocyte sedimentation rate (ESR) and C-reactive protein (CRP), are documented to be increased. CAP is further linked with sepsis. Low blood pressure, altered mental status, and different signs of organ malfunction, such as renal dysfunction, liver problems, and/or thrombocytopenia, are some primary characteristics of pneumonia [115].

It is now generally acknowledged that TNF-α plays a critical mediating role in dictating the course of a wide range of infectious diseases in the host [116]. Complex interactions between immune cells and both pro-inflammatory and anti-inflammatory cytokines are essential for the regulation of the inflammation that occurs in bacterial pneumonia [117]. Tumor necrosis factor α (TNF α) and interleukin 1 (IL-1) are two crucial pro-inflammatory cytokines and early response mediators that activate innate immune response [118,119]. In addition to host defense and inflammation, TNFR2 activation is linked to homeostatic bioactivities such as tissue regeneration, cell proliferation, and cell survival [119]. A competitive occupancy of the IL-1 receptor without agonist action is caused by the interleukin 1 receptor antagonist (IL-1ra). A rise in anti-inflammatory cytokines in CAP may be attributed to PMN. Strategies to boost neutrophil numbers may have positive benefits by altering the inflammatory cytokine response as well as enhancing the antibacterial activity [85].

## 6. Types of Bacterial Pneumonia

Pneumonia can be classified into two types based on how the infection is acquired. However, histopathologically, pneumonia can be categorized as lobular, lobar, bronchopneumonia, or interstitial depending on the part of the lung affected. A descending infection that began around the bronchi and bronchioles and then locally moved to the lungs is known as bronchopneumonia. Usually, lower lobes are affected. Patchy consolidation patches in the bronchi and alveoli indicate neutrophil accumulation. Acute exudative infection of the whole lobe is known as lobar pneumonia [11].

### 6.1. Community-Acquired Pneumonia (CAP)

Community-acquired pneumonia (CAP), which is an important cause of morbidity and mortality worldwide, can be described as an acute infection of the pulmonary parenchyma obtained outside of a hospital. CAP may present medically in a number of ways, from mild pneumonia with a persistent cough and fever to severe pneumonia with sepsis and respiratory failure. CAP is included in the differential diagnosis of almost all respiratory infections because of the broad range of related clinical symptoms [120].

### 6.2. Nosocomial Pneumonia

Including both ventilator-associated pneumonia (VAP) and hospital-acquired pneumonia (HAP), it is an acute infection of the lung parenchyma that develops in medical facilities [121].

#### 6.2.1. Hospital-Acquired Pneumonia (HAP)

It is an acute infection of lung tissue in a non-intubated patient that develops after 48 h of hospitalization [23]. In other words, a lower respiratory infection that was not incubating at the time of hospital admission and that manifests clinically two or more days after hospitalization is known as hospital-acquired pneumonia (HAP) (https://emedicine.medscape.com/article/234753-overview?form=fpf, accessed on 3 October 2024).

#### 6.2.2. Ventilator-Associated Pneumonia (VAP)

It is a specific type of nosocomial lung infection that often appears 48 h or more after an intubation for mechanical ventilation. The second most typical hospital-acquired disease among pediatric and neonatal critical care unit patients is ventilator-associated pneumonia. In total, 10% of all pediatric device-related infections and 7% to 32% of healthcare-associated infections attributed to it have been reported to the National Healthcare Safety Network (NHSN). In general, pediatric intensive care units (PICUs) have a lower rate of pneumonia than adult critical care units (ICUs). The rate of ventilator-associated pneumonia in newborns is inversely related to birth weight [122].

#### 6.2.3. HCAP (Healthcare-Associated Pneumonia)

Furthermore, patients receiving care services for healthcare-associated pneumonia (HCAP) fall into a specific pneumonia category. These patients include those in a long-term care facility, those hospitalized in an acute care hospital for two or more days within 90 days of the infection, those who recently received intravenous antibiotic therapy, those who received chemotherapy in last 30 days, or those on hemodialysis. Patients diagnosed with HCAP, as opposed to the other types of pneumonia, might benefit from distinct causing microorganisms and antibiotic resistance profiles. On the other hand, the initial HCAP management is currently equivalent to that of CAP or HAP [123],

#### 6.2.4. Aspiration Pneumonia

Aspiration pneumonia arises when particulate matter, stomach content, or oropharyngeal secretions, which are rich in bacteria, are inhaled into the lower respiratory tract. It primarily affects older persons and presents a serious risk for morbidity and mortality, especially in those with advanced age, limited mobility, frailty, and underlying comorbidities. A higher risk also applies to people with learning difficulties and gastrointestinal and neurological conditions that impair normal swallowing function. The majority of cases of aspiration pneumonia in the elderly population are brought on by complex patient background factors [124] Frailty and underlying long-term medical problems are strongly associated with the diagnosis of aspiration pneumonia in older adults [125].

## 7. Pathophysiology of Pneumonia

The essential function of delivering oxygen to every cell in the body is carried out by the lungs, which are made up of an integrated network of terminal airways referred to as the terminal bronchioles. Several respiratory cell types, such as respiratory epithelial cells, endothelial cells, various stromal cells, and alveolar macrophages, as well as other organ-specific cells that frequently occur in the terminal structures of each organ, make up the terminal structures (https://www.nhlbi.nih.gov/health/lungs/respiratory-system, accessed on 3 October 2024). As a result, the etiology of ARDS, also known as acute respiratory distress syndrome, is most effectively described as an important acute injury produced directly through several insults, such as infectious agents and/or host immunological barrier responses, to a specific kind of respiratory cell [126].

To produce pneumonia, the virus must either overpower the host’s already compromised immune system or enter the alveolar space and have a large enough inoculum. The macrophages start an inflammatory reaction as a result of the pathogen’s unchecked growth in the alveolar gaps [127]. Nearly every cell produces cytokines, which are tiny, secreted proteins (<40 kDa) that control and affect the immune response. Pro-inflammatory cytokines will cause the immune system to be activated, produce more cytokines, and release more of them [128]. The clinical signs and symptoms of pneumonia are brought on by cytokines that are generated as a result of the inflammatory response. For instance, whereas tumor necrosis factor (TNF-α) and interleukin-1 (IL-1) generate fever, interleukin-8 (IL-8) and colony-stimulating factors cause chemotaxis and neutrophil maturation, which results in leukocytosis. The alveolar–capillary membranes leak as a result of this inflammatory response, reducing lung compliance and causing dyspnea [13]. Alveolar damage is the result of this, and alveolar–capillary membrane leakage, edema, and microhemorrhage are the results of a cell-mediated inflammatory response [129].

## 8. Diagnosis of Pneumonia

A solid physical examination and a well-collected history form the basis of a diagnosis of pneumonia. In order to determine the reason of immunodeficiency, the general questionnaire can be provided to patients regarding their sexual habits and any history of cancer, organ transplantation, corticosteroid usage, or any disease requiring chronic immunosuppressive medication. The assessment and diagnostic approaches for pneumonia can be divided into three categories [127].

### 8.1. Common Laboratory Examination

#### 8.1.1. Complete Blood Count (CBC) and Differential Leucocyte Count

If the cause of the infection is bacterial, leukocytosis as well as an elevated neutrophil count may be present, assuming the patient does not already have immunodeficiency brought on by neutropenia. The leukocyte count may be increased, reduced, or normal in viral pneumonia. In 2015, 95 patients diagnosed with CAP and under the age of 18 participated in this prospective descriptive research. Data on each patient’s clinical, laboratory, and chest radiography findings were gathered. When CAP patients were evaluated at the time of admission, platelet count was suggested to be a more useful indicator of severity and outcome when examining CBC values [130]. A recent study showed that red blood cell distribution and lymphocytes were the most useful predictors of disease severity identifying HIV-infected patients with CAP who required ICU admission [131]. In a prospective study of CAP patients, C-reactive protein, procalcitonin, proadrenomedullin, copeptin, white blood cells, lymphocyte count percentage (LCP), neutrophil count percentage (NCP), and neutrophil–lymphocyte ratio (NLR) were measured in blood tests performed at the time of admission and during the early stages of the disease (72–120 h). Procalcitonin, copeptin, and proadrenomedullin levels were higher in patients who died during follow-up, while LCP levels were lower and NCP and NLR levels were higher. The results suggest that NLR and NCP might be good diagnostic markers [132].

#### 8.1.2. Pro-Inflammatory Cytokines

Ongoing inflammation will cause inflammatory indicators, including C-reactive protein (CRP), erythrocyte sedimentation rate (ESR), and procalcitonin, to increase. Procalcitonin and CRP can also aid in early-stage pneumonia prognosis prediction and pneumonia severity. Pneumonia outcomes can be complicated by inflammation. The invasion of the respiratory pathogen necessitates an efficient and prompt inflammatory response. On the other hand, even in patients receiving top-notch medical treatment, a toxic and persistent inflammatory response can cause lung harm and poor results [133]. In a study conducted on 346 patients, ESR information was gathered for 278 patients. ESR was documented to be more than 20 mm/h in 80.2% of the patients, with a median value of 53 [134]. In a prospective study conducted on children with clinical and radiographic pneumonia admitted at children’s hospitals from 2014 to 2019 who were 2 months to 18 years old, it was documented that procalcitonin levels rose along with severity and length of stay (LOS). As a conclusion, increased severity and LOS were connected with higher procalcitonin levels [135]. In a cohort study of 1886 patients admitted with CAP to 28 academic and community hospitals in the US, the levels of plasma TNF-α, IL-6 (interleukin 6), and IL-10 were evaluated every day for the first week and once a week after that. The combination of high levels of pro-inflammatory IL-6 and anti-inflammatory IL-10 cytokine activity was found to be associated with the highest risk of mortality [136]. Between September 2008 and February 2011, 862 children who were hospitalized for acute respiratory tract infections were assessed; the serum levels of CRP were tested in each kid. Serum CRP levels were found to be significantly correlated with severe pneumonia and bacterial infections. Therefore, the CRP level and other variables were suggested to be used as early warning signs of the onset of severe pneumonia [137].

#### 8.1.3. Chemistry Panel

The patient’s level of hydration will be determined by the results of a chemical panel.

#### 8.1.4. Sputum Smear and Culture

To understand the causes of various lung and airway diseases, sputum examination is crucial. The fluid secreted by the lungs and airways, also known as the bronchial and windpipes, is known as mucus. Sputum is the precise name for the mixture of saliva and mucus that is specifically coughed up from the respiratory tract, frequently after an infection or an irritant of the mucosa. If at all possible, a sputum smear and culture should be acquired. The first stage of laboratory sputum analysis is smear microscopy. It is a quick and affordable technique that works well in environments with limited resources. The smear sputum slide is stained with different dyes according to the instructions. Then, the pathology specialist examines the stained slide under the microscope to find the abnormal cells from the sputum specimen [138]. Gramme stain is used to distinguish between the two main categories of bacteria, gram-positive and gram-negative microorganisms [139]. For the purpose of identifying fungi, the Grocott–Gomori’s methenamine silver stain (GMS) is a widely used staining technique. In order to recognize *Pneumocystis jirovecii*, GMS staining is essential [140,141].

The next step in identifying the bacteria is to conduct biochemical tests of bacterial growth. Motility, McFarland standard, fluid thioglycollate medium (FTM), catalase, and oxidase tests are typical biochemical procedures used to detect bacterial growth. A substance specifically designed to encourage the growth of bacteria or fungi is added to a culture plate with the sputum sample. Microscopy, colony morphology, or biochemical tests of bacterial growth will be carried out after the sputum culture is positive in order to find the precise bacterium or fungus. To identify a suspected organism, at first, the bacteria will be inoculated in a series of differential media. Then, different indicators are used to observe the specific endproducts of metabolism inside of the medium [138].

#### 8.1.5. Bronchoalveolar Lavage (BAL)

The minimally invasive treatment known as bronchoalveolar lavage, or BAL, involves injecting sterile normal saline into a subsegment of the lung, followed by suctioning the instillation out and collecting it for testing. A flexible bronchoscope is often inserted into a lung subsegment to facilitate this surgery. The procedure became widely known in 1974 because of the efforts of American doctors Reynolds and Newball in Maryland. Today, it is primarily used as a diagnostic technique to assess lower respiratory tract pathology, while in select rare cases, it can also be used therapeutically [142].

Differential BAL cell counts have been described as being frequently indicative of particular lung diseases. Additionally, more specialized diagnostic procedures have been described as part of BAL fluid analysis. These procedures include molecular assays like polymerase chain reaction (PCR) or enzyme-linked immunosorbent assay, unique cytopathologic stains, or distinctive microscopic findings [143].

#### 8.1.6. Blood Culture

Due to the perceived higher risk of bacteremia, particularly with multidrug-resistant organisms, blood cultures are of little value in non-severe CAP but are frequently advised for severe community-acquired pneumonia or pneumonia linked with hospitalization. This procedure has no recognized benefit [144]. Before starting any antibiotics, blood cultures should be performed according to the rule. Blood cultures are increasingly being used as a diagnostic tool due to advancements in both methods and outcomes over time. When blood cultures are positive and there is a chance of contamination, the diagnostic conundrum appears. Therefore, taking blood cultures in the proper environment and having a hospitalist interpret them are essential to managing the hospitalized patient [145].

#### 8.1.7. CD4 Counts

Bacterial pneumonia can occur at any stage of HIV disease and at any CD4 cell count. However, as the CD4 cell count declines, the incidence of bacterial pneumonia increases, as does the incidence of accompanying bacteremia and septicemia. The latter is especially the case with *S. pneumoniae*. The clinical presentation of HIV-associated bacterial pneumonia is similar to that in persons without HIV infection. Persons with bacterial pneumonia typically present with the acute onset (3 to 5 days) of fevers chills/rigors, chest pain, dyspnea, and cough that is productive of purulent sputum. Lung examination reveals evidence of consolidation and occasionally pleural effusion. Laboratory testing is usually notable for an elevated white blood cell count, often with a predominance of polymorphonuclear leukocytes (PMNs) [146].

### 8.2. Chest X-Ray (CXR)

The chest X-ray (CXR) is a simple, affordable, and widely used method for identifying lung conditions. An expert radiologist can determine if an X-ray is normal or indicates a condition such as lung cancer, TB, or pneumonia [147,148]. When a suspected case of pneumonia is being examined, this is the first-line imaging technique of choice. Radiographs of the posterior anterior (PA) and lateral planes are taken. In patients with immune-compromised conditions, CXR imaging may be normal for up to 72 h despite symptoms.

### 8.3. Computer Tomography (CT)

Despite being the most popular diagnostic tool, the chest X-ray (CXR) has severe shortcomings. In polymorbid patients or elderly individuals who are bedridden, obtaining high-quality pictures is difficult. With k coefficients ranging from 0.37 to 0.53 for the presence of pneumonia, CXR has poor sensitivity and interobserver agreement [149]. As a result, the use of biomarkers of inflammation or infection, such as procalcitonin and C-reactive protein (CRP), as a guidance in the diagnostic process has been recommended. Almost all infectious, autoimmune, ischemic, and neoplastic disorders can lead to a rise in blood CRP levels. All multimorbid patients (65 years and older) were admitted to an internal medicine hospital ward in Italy from January to August 2013 and were analyzed using a retrospective cohort study design. Each patient’s pneumonia diagnosis, comorbidities as represented by the Cumulative Illness Rating Scale (CIRS), living situation, length of stay, serum hs-CRP, and procalcitonin levels at admission were recorded. As per the conclusion of this cohort study, when clinical suspicion of pneumonia exists in older multimorbid individuals who require hospitalization for respiratory symptoms, serum high-sensitive hs-CRP testing appears to be more beneficial than procalcitonin in guiding the diagnostic process. Procalcitonin testing may thus be contraindicated in this situation [150].

A CT scan is highly recommended, since CXR tends to identify only a few abnormalities in neutropenic patients. The CT scan lowers pneumonia overdiagnosis and enables improved identification of alternate diagnoses. The influence on clinical outcomes of a strategy incorporating CT scans for patients suspected of pneumonia, as well as its cost-effectiveness, should be assessed [149]. Claessens et al. showed that in 319 patients who visited the emergency room with a possible diagnosis of CAP, an early CT scan reduced the likelihood of the condition in 100 patients (31%) [151]. Prendki et al. showed that in 200 elderly individuals with suspected pneumonia, a low-dose CT scan (LDCT) reduced the likelihood of pneumonia in 54 patients (27%) [152]. 

### 8.4. Additional Techniques

#### 8.4.1. Legionella and Pneumococcus Urine Antigen Testing

Urinary antigen testing (UATs) for *Streptococcus pneumoniae* and *Legionella pneumophila* are indicated in adult community-acquired pneumonia (CAP) guidelines from the Infectious Diseases Society of America (IDSA) and American Thoracic Society (ATS). The sensitivity and specificity of the IDSA/ATS UAT indications for identifying patients who test positive were assessed using data from a multicenter, prospective, surveillance study of people hospitalized with CAP. Among 1941 patients, 81 (4.2%) had SP-positive UATs and 32 (1.6%) had LP-positive UATs. Future CAP guidelines should take other methods into consideration for selecting patients who should undergo urinary antigen testing because the IDSA/ATS CAP guidelines’ suggested indications for SP and LP urinary antigen testing have low sensitivity and specificity for identifying patients with positive tests [153].

#### 8.4.2. ELISA and PCR

Children frequently contract respiratory tract infections caused by *Mycoplasma pneumoniae*, which presents a clinical barrier for accurate and prompt diagnosis. For the detection of this infection, real-time polymerase chain reaction (RT-PCR) has been utilized often. *Mycoplasma pneumoniae* infection is often diagnosed using serology, which can take up to two weeks for the development of diagnostic antibodies. Methods based on PCR enable earlier diagnosis. For diagnosing MP infection in its early stages, PCR is more accurate than serology [154]. Infections with Mycoplasma emerge as epidemics (every 3–7 years) and are frequently the cause of CAP in individuals who have been in close contact for an extended length of time, particularly in children, adolescents, and the elderly. Mycoplasma infections have long been routinely diagnosed using ELISA techniques, which measure the presence of certain Mycoplasma antibodies of classes G, M, and A in serum. PCR, a new diagnostic technology that finds Mycoplasma’s DNA in clinical samples, is one of the most trustworthy strategies [155].

#### 8.4.3. Beta-D-Glucan Testing

A helpful tool for supporting a quantitative PCR (qPCR)-based diagnosis of suspected *Pneumocystis pneumonia* (PCP) with bronchoalveolar lavage (BAL) fluid is serum beta-d-glucan (BDG) measurement [156]. BDG is a structural polysaccharide formed during the synthesis of fungal cell walls in many pathogenic fungi, and it may be detected using assays that were created and authorized largely for the diagnosis of invasive candidiasis and pulmonary aspergillosis [157].

### 8.5. Lung Ultrasound

Since lung ultrasound (LUS) is used to assess and measure the quantity of B-lines, pleural abnormalities, and nodules or consolidations, it has been shown to be a new non-invasive bedside method for diagnosing interstitial lung syndrome. According to reports, LUS is a promising diagnostic and follow-up tool for both adult and pediatric pneumonia [158]. LUS is more accurate than chest CT scans at diagnosing pneumonia [159]. However, LUS has been considered to be equal to CT scans for interstitial lung syndrome (interstitial pneumonia, interstitial disorders, and ARDS) [158].

LUS shows the typical bilateral pattern of diffuse interstitial lung syndrome in patients with COVID-19 pneumonia. This pattern includes thickening of the pleural line with pleural line irregularity, multiple or confluent B-lines with spared areas, and, less frequently, subpleural consolidations and pleural effusion [158,160]. Another study finding shows that LUS was a potential method for detecting COVID-19 pneumonia in patients who visited the emergency department (ED) during an active outbreak [161].

Individuals with or without COVID-19 pneumonia can be quickly identified by combining LUS patterns of probability with clinical characteristics at presentation [162]. In a study, the pulmonary ultrasonography patterns of COVID-19 patients were examined after their admission and discharge from the intensive care unit. The lateral and posterior non-translobar C and B2 patterns were the most common features of LUS obtained from COVID-19 patients who had severe respiratory failure during ICU admission and discharge. In both the survived and non-survived patient groups, the computed LUS score remained high at discharge with no discernible change from admission [163]. Further, lung ultrasound score was a strong predictor of death, intensive care unit admission, and endotracheal intubation in COVID-19 patients who were admitted to the emergency department [164].

## 9. Biomarkers for Detection of CAP

A more reliable and unambiguous test is required for distinguishing between viral and bacterial pneumonia. In this context, the study of biomarkers that enter a patient’s bloodstream throughout the development and course of the disease appears to be a promising avenue. Given the numerous confounding variables that must be taken into account for interpretation, the measured levels of biomarkers should always be associated with clinical results and interpreted with caution [165]. Recently, various inflammatory biomarkers such as procalcitonin (PCT), soluble triggering receptors expressed on myeloid cells-1 (sTREM-1), proadrenomedullin (proADM), and presepsin have been identified as relatively specific biomarkers for bacterial infection [166]. Numerous biomarkers are listed in Table 2.

The CALC-I gene on chromosome 11 encodes the protein procalcitonin (PCT), which undergoes multiple post-translational modifications to yield calcitonin and several other free peptides. An elevated risk of bacteremia, septic shock, multi-organ failure, and mortality was linked to PCT levels more than 2 ng/mL. CRP levels below this threshold could rule out a diagnosis of confirmed CAP. A cut-off value of 33 mg/L CRP allowed for 83% sensitivity and 44% specificity in separating patients with a confirmed diagnosis of CAP from those with comparable clinical symptoms but distinct clinical diseases [173]. Because it induces IL-2, which improves T-cell development, IL-6 has a role in a number of hematological, immunological, and inflammatory responses [165]. Studies have demonstrated a strong association between IL-6 levels and several clinical severity scores, including MEWS, CURB 65, and the pneumonia severity index (PSI) [174].

The purpose of the high-affinity immunoglobulin Fc γ receptor on neutrophils, or nCD64, is to enable these cells to opsonize and phagocytose pathogens [165]. More clinical deteriorations and ICU admissions were seen in individuals whose nCD64 expression was greater than or equivalent to 2700 mean fluorescence intensity (MFI). The low sensitivity of 44.4% for clinical deterioration and 33.3% for ICU admission and the specificity of 90.1% and 90.8%, respectively, were seen in these patients [175]. Patients with severe community-acquired pneumonia (CAP) have been reported to have elevated D-dimer levels [176].

In numerous disorders, circular RNAs (circRNAs) have been shown to be useful biomarkers for both diagnosis and treatment. A study using microarray profiling analysis found 8296 circRNAs that were differentially expressed (DE) in CAP patients (*n* = 6) compared to healthy controls (*n* = 6). In addition, the study predicted 205 mRNA target genes and found 10 miRNAs that were most likely to interact with these four circRNAs. Highly likely functional consequences were shown by the KEGG pathway enrichment study in relation to inflammation and signaling pathways associated with viral infections [177]. Gene expression patterns that were both common and unique across CAP and non-CAP patients were identified by blood microarray research. The FAIM3:PLAC8 ratio was proposed as a potential biomarker to help with the quick diagnosis of CAP upon admission to the intensive care unit [178]. MiRNA biomarkers for pneumonia were identified by RNA sequencing and bioinformatics analysis. Several important DE-miRs, including hsa-miR-200b, hsa-miR-455, and hsa-let-7f-1 in pneumonia, were discovered in a recent study [179].

Myxoma resistance protein (MxA1) is a protein that tends to increase greatly after viral rather than bacterial infection, in comparison to other indicators. MxA1 can be activated by type I or type III interferon (IFN), but not by the type II IFN signaling pathway or by direct bacterial or viral contact [180]. MxA1 > 200 ng/mL and low CRP (<40 mg/L) are strongly suggestive of a viral etiology [181]. Binding to DNA, high-mobility group box one protein (HMGB1) triggers the transcription of several inflammatory markers. Moreover, it plays certain extracellular functions like stimulating migration and raising the synthesis of pro-inflammatory cytokines and indicators like interleukin 6 (IL-6), tumor necrosis factor (TNF), or interferon gamma (IFN-γ). This protein is increased in sepsis, CAP, and viral-bacterial co-infections, particularly when the influenza virus and bacteria are co-infected [182]. In addition, other biomarkers, including endotoxin, cortisol, and endothelin-1 precursor peptides, are also being used in the detection and diagnosis of pneumonia [183].

Presepsin is a fragment of the monocyte lipopolysaccharide (LPS) receptor CD14. A novel biomarker for sepsis and death in community-acquired pneumonia is the serum presepsin level. A study showed that serum presepsin levels were significantly correlated with procalcitonin, lactate, C-reactive protein, pneumonia severity index, and fast sequential organ failure assessment (qSOFA) [184]. When bacteria are phagocytosed, presepsin is released into the bloodstream. When assessed in a combined evaluation, high levels of presepsin appear to predict the progression to septic shock and severe CAP, improving the predictive and diagnostic accuracy of other markers, like PCT [185].

D-dimer is a commonly used, readily measurable biomarker for thromboembolic diseases and is a byproduct of fibrin decomposition. The pathogenic function of fibrinolysis and coagulation in the development of acute lung injury is reflected in blood levels of D-dimer. According to reports, patients with severe CAP have higher D-dimer levels, which are linked to a higher risk of death [165]. The findings of a recent study show that D-dimer levels were considerably elevated in both COVID-19 and CAP patients upon admission, with COVID-19 patients having higher D-dimer levels than CAP patients. Additionally, correlation between D-dimer and inflammatory markers was noticed in COVID-19 patients [186]. In 2021, retrospective analysis was published on the clinical, imaging, and laboratory data of 120 patients who had an RT-PCR-based COVID-19 diagnosis. Of the patients, 63.3% (76/120) had elevated D-dimer. Age, length of stay, lung involvement, fibrinogen, neutrophil count, neutrophil lymphocyte ratio (NLR), and platelet lymphocyte ratio (PLR) all had favorable associations with D-Dimer readings [187].

In many clinical conditions, matrix metalloproteinases (MMPs) play a role in tissue degradation. When MMPs are active in the lung, bioactive mediators with inflammatory qualities are released. In the lungs of patients with non-survival COVID-19, MMP-2 and MMP-8 expression were elevated and correlated with the release of sTREM-1 and sHLA-G. Along with neutrophil infiltration and its byproducts, such as reactive oxygen species (ROS), the overexpression of the MMP-2/MMP-8 axis also boosted lipid peroxidation, which may facilitate the severe loss of lung tissue in COVID-19 [188]. MMP3, or matrix metalloproteinase 3, is referred to as an inflammatory factor. In a study, Wang and colleagues assessed serum MMP3’s diagnostic and predictive utility in pneumonia patients. The findings show that pneumonia patients had considerably greater admission amounts of MMP3, NGAL, and IL-6 than healthy controls. Therefore, MMP3 is a useful biomarker for determining the severity of pneumonia and forecasting mortality in those patients [189].

A 25 kDa protein called neutrophil gelatinase-associated lipocalin (NGAL) possesses chemostatic and bacteriostatic properties, aids in iron transport, and is linked to acute kidney damage. Both neutrophils and respiratory epithelial cells actively produce this protein. The purpose of a study was to find out if plasma NGAL levels may predict in-hospital mortality and admission to the intensive care unit (ICU) in patients with pneumonia. The findings imply that the plasma NGAL level is a valuable biomarker for forecasting hospitalized pneumonia patients’ ICU admission and death [190]. Investigating the predictive significance of urine neutrophil gelatinase-associated lipocalin (uNGAL) in relation to death in hospitalized COVID-19 patients was the goal of one investigation. According to the study’s findings, uNGAL concentration in COVID-19 patients is correlated with disease outcome [191].

## 10. Next-Generation Sequencing and Its Application in Pneumonia Diagnosis

In genomics research, next-generation sequencing (NGS) is an effective technique. Millions of DNA fragments can be sequenced simultaneously by NGS, yielding comprehensive data on genome structure, genetic variants, gene activity, and behavioral changes in genes. The main goals of recent developments have been to improve data analysis, lower expenses, and sequence data more quickly and accurately. These developments have enormous potential to improve our understanding of diseases and tailored therapy by revealing fresh insights into genetics [192].

The advancement of next-generation sequencing (NGS) has improved the efficacy of pathogen detection, and it is crucial to precisely identify the underlying causes of infectious disorders such severe pneumonia. A total of 130 patients treated in the intensive care unit (ICU) between June 2022 and June 2023 for severe pneumonia were the subject of a retrospective investigation. Over 70% of the tNGS results for the analysis of the 130 individuals matched the findings of the clinical diagnostic tests. The results of culture, mNGS, and RT-qPCR were consistent with the pathogenic bacteria detected by tNGS [193]. Using metagenomic next-generation sequencing (mNGS), the etiological spread of pediatric refractory pneumonia was examined. A total of 43 specimens from the 60 children with refractory pneumonia tested positive for 67 pathogen strains using mNGS. The findings show that mNGS is more effective overall than conventional techniques in determining the cause of children’s refractory pneumonia [194]. A recently discovered technique to swiftly, effectively, and impartially gather information on microbial nucleic acid sequences is called metagenomic next-generation sequencing (mNGS). In a prospective trial involving 138 patients from a single hospital, shotgun metagenomic next-generation sequencing (mNGS) of bronchoalveolar lavage fluid (BALF) was used for pathogen identification in pneumonia [195]. In children with severe nonresponding pneumonia, metagenomic next-generation sequencing (NGS) can improve the sensitivity of pathogen detection. Furthermore, mass spectrometry (mNGS) will provide more strain-specific data, aid in the identification of novel diseases, and may even aid in the tracking and management of epidemics [196].

When compared to conventional diagnostic techniques, metagenomic sequencing increases the effectiveness of pathogen identification and can be used in clinical diagnosis. In order to identify new, uncommon, and unexpected diseases, metagenomic sequencing may be useful. Additionally, it can identify co-pathogens and offer thorough pathogen information [197]. A report showed that the detection rate of mNGS for pathogens was greater as compared to standard BALF culture in CAP patients. Further, mNGS can identify bacteria more quickly and accurately [198]. A study was conducted to compare the advantage of metagenomic NGS with traditional pathogen detection techniques. The findings show that mNGS detected at least one microbial species in nearly 89% of pulmonary infection patients. Additionally, mNGS detected human disease-related microbes in 94.49% of pulmonary infection patient samples that had yielded negative results from traditional pathogen detection. The accuracy and sensitivity of mNGS were reported to be higher than those of traditional pathogen detection. Moreover, findings show that mNGS simultaneously detected and identified a wide range of pathogens [199]. With a detection sensitivity that exceeds that of traditional culture techniques, the tNGS methodology was reported to have quick and efficient capabilities in identifying bacteria, fungi, viruses, and particular diseases [111]. In patients with suspected blood stream infections, NGS-based diagnostics may provide a greater positive rate than traditional culture-based techniques. However, given their current high cost, their influence on anti-infective therapy is currently restricted. Therefore, before NGS can be implemented as a standard test in infectious disease diagnosis, bigger randomized multicenter trials must show their potential positive influence on patient outcomes [200]. Further, mNGS has been found to be less specific as compared with traditional techniques [199]. In brief, the application of NGS faces several challenges, including cost, turnaround time, technical complexity, correlation to diseases, processing of samples, etc.

Early detection of hospital and ventilator-associated lung infections is essential due to the rise in multidrug-resistant microorganisms. This allows for proper treatment of patients from the beginning without the need for broad-spectrum antibiotics, which may not be effective against the underlying cause. The PCR method was the most rapid and accurate diagnostic method until recently. POC-PCR is the most suitable technique for identifying microorganisms that cause VAP and any genes linked to antibiotic resistance, since it allows for quick and easy sample analysis without the requirement for transportation or intermediary preparatory steps [201]. Compared to conventional culture methods, molecular diagnostic testing was able to identify 23.6% more infections [202]. Additionally, comprehensive molecular testing only needs one LRT sample and greatly enhances pathogen detection in CAP, especially in patients treated with antibiotics. Additionally, it may make it possible to switch from broad-spectrum empirical antimicrobials to pathogen-directed therapy early on. Further, sputum PCR testing was not enough on its own. Nonetheless, a major problem is the absence of positive microbiological identification [203]. The merits of these quick syndromic panels include their high sensitivity, specificity, and negative predictive value when used in conjunction with expert interpretation, but they should not replace conventional culture and antibiotic susceptibility tests. The drawbacks include false-positive results because they detect nucleic acids from dead bacteria that are not generating an active infection, lack of detection for off-target infections, lack of complete susceptibility information, and expense [204].

## 11. COVID-19 and Pneumonia

Coronavirus disease 2019 has been considered to be a dynamic disease and is caused by severe acute respiratory syndrome coronavirus 2 (SARS-CoV-2) [205,206]. A significantly high rate of subsequent bacterial infection was seen in critically ill patients infected with SARS-CoV-2 [207]. High rates of intensive care unit admission and in-hospital mortality are linked to COVID-19 pneumonia [208]. A study suggested that pregnant women with COVID-19 should be continuously monitored, and the risk factors that influence mortality should be identified in order to make the appropriate preparations [209]. Glucocorticoids, antiviral medications, and the JAK inhibitor baricitinib are used to treat severe pneumonia. Baricitinib works by inhibiting viral receptor-mediated endocytosis, which is mediated by the NF-κB activating kinase (NAK) family, and by inhibiting JAK 1/2, which mediates the anti-cytokine actions. It lowers mortality and improves severe pneumonia. Therefore, the identification of molecularly targeted drugs with clear pathogenic mechanisms could help in managing COVID-19 infection [210].

According to a study, patients with COVID-ARDS had different epidemiology and important ventilation characteristics to patients with CLASSIC-ARDS. Additionally, patients with COVID-ARDS had lower ΔP [211]. To determine if the severity and mortality risk factors for patients hospitalized for COVID-19 pneumonia differed between the early wave and the very late stage of the pandemic, a study was published. The results of these investigations show that there may be differences in the clinical signs, severity, and mortality risk factors of COVID-19 between the early wave and the very late stage of the pandemic [212]. Following COVID-19, patients with pneumonia must have a reevaluation that includes a chest CT scan; some of these patients may be eligible for an early lung biopsy. The most persuasive and successful treatment for organizing pneumonia caused by COVID-19 is corticoid therapy at a dosage equal to 0.5 mg/kg/day of prednisone [213].

The clinical manifestations of COVID-19 can vary greatly over time, from people with no symptoms to those experiencing severe respiratory failure [214]. The symptoms of many COVID-19 patients who need to be hospitalized resemble those of community-acquired bacterial pneumonia, which leads to the empirical use of antibiotics. Experience with hospitalized influenza patients, of whom 11% to 35% may develop a bacterial superinfection, may potentially contribute to the high use of antibiotics [207]. According to several studies, patients treated with nirmatrelvir/ritonavir (Paxlovid) during the acute phase of COVID-19 have a lower chance of developing severe COVID-19 disease and post-COVID-19 conditions. Follow-ups at 4–6 weeks and 12 weeks following discharge are recommended for the pulmonary care of inpatient COVID-19 cases. Chest imaging, pulmonary embolism screening, 6 min walk tests, and pulmonary function testing should be used to evaluate patients who have chronic dyspnea or who need extra oxygen (https://emedicine.medscape.com/article/2500117-overview?form=fpf, assessed on 14 November 2024). However, the majority of COVID-19 patients in intensive care units need breathing support. It is considerable that treatment for ARDS and pneumonia is essential during times of high mortality, but the new approach to pneumonia management needs to be reconsidered.

By inducing anti-inflammatory cytokine responses that counteract pro-inflammatory cytokines, low-dose radiation treatment (LD-RT) may offer therapeutic benefits against morbidity and mortality rate linked with COVID-19. In addition to offering a therapy strategy for COVID-19-associated pneumonia, an investigation study conducted by Houssien sought to explore the application of low-dose lung radiation in cases of bacterial and viral pneumonia. With fewer adverse effects, the administration of LD-RT at different stages of COVID-19 seems to be beneficial [215].

The purpose of a study was to ascertain whether non-ICU septic patients with and without type 2 diabetes (T2D) had different primary outcomes. In septic patients who do not require intensive care unitization, DM does not seem to have a detrimental effect on outcomes [216]. A multicenter prospective cohort study of T2D patients hospitalized with COVID-19 was conducted. A higher risk of in-hospital death, intensive care unit (ICU) admission, and progression to acute respiratory distress syndrome (ARDS) was found to be linked with the use of DPP4 inhibitors for the chronic management of type 2 diabetes [217].

## 12. Association Between HIV Infection, Corticosteroid Treatment for Rheumatic Disease, and Diabetes and Pneumonia Infection

Diabetes mellitus is a prevalent metabolic condition with proven negative consequences on immunity, and patients with diabetes mellitus have impaired phagocytosis and macrophage chemotaxis, impaired T-cell anergy as evidenced by delayed hypersensitivity skin tests, and a poor lymphoproliferative response to mitogens due to chronic hyperglycemia exposure. Therefore, diabetes patients are more vulnerable to infection such as bacterial and fungal respiratory tract infections, as well as systemic viral diseases [218]. An increased risk of T2D is one potential side effect of various highly active antiretroviral (HAART) drugs used by HIV patients. Exposure to HAART greatly increases the risk of diabetes in addition to the age-related increase. Certain anti-HIV drugs may increase the risk of diabetes [219].

Additionally, diabetes is more common among HIV-positive individuals than in the general population. Nonetheless, many HIV-positive individuals have some of the risk factors for diabetes, such as weakened immune system, impaired lung function, increased risk of aspiration, coexisting comorbidities such as cardiovascular diseases, etc. [220]. Immune responses can be negatively impacted by extrinsic causes, leading to secondary immunodeficiency and an elevated risk of infection. These immunodeficiencies are caused by a variety of factors, including glucocorticoid and immunomodulatory medication treatment, trauma and surgery, harsh environmental circumstances, and long-term infections, including HIV-related ones [218].

Glucocorticoids are used extensively to reduce inflammation, particularly during the acute phase. Despite their effectiveness, there are a number of well-documented adverse effects linked to prolonged use or high dosages of them [221]. Rheumatoid arthritis (RA) is a chronic systemic inflammatory disease, and the most common cause of death and morbidity among RA patients is cardiovascular disease (CVD). RA patients may be at higher risk of DM, particularly among those receiving glucocorticoids (GCs), while patients on hydroxychloroquine and biological disease-modifying anti-rheumatic treatments (DMARDs) may be at lower risk [222].

One of the most prevalent is hyperglycemia, often known as frank diabetes, which is thought to affect 10–20% of people. The primary cause of its pathophysiology is elevated insulin resistance [223]. Advanced glycation end-stage products (AGEs) and oxidative stress markers may be contributing causes to low-grade inflammatory pain in diabetic people with osteoarthritis (OA). Uncontrolled glycemic status in obese people with type 2 diabetes can result in oxidative damage, ligament calcification, and ossification brought on by mechanical stress [224].

## 13. Conclusions

Serious respiratory tract infections, including pneumonia, are more likely to occur in people with diabetes. The hospital admission and mortality rates of diabetes patients are higher compared with non-diabetic patients. Poor pneumonia care and a lack of understanding about how to lower hospital admission and pneumonia patient mortality persist despite the significance of pneumonia for human health and the fact that many of the investigations mentioned have been areas of focus for a number of years. Better knowledge of risk factors, causes of infection, pathomechanisms, and early identification or clinical predictors can reduce the hospital admission and mortality rates due to pneumonia in diabetic patients. The application of diagnostic tests to determine the causes of community-acquired pneumonia is limited since there are currently no quick, precise, easy-to-use, or economical methods for determining the factors and for confirmation of pneumonia infection. A new direction in medicine has been brought about by the variety of biomarkers and molecular techniques for identifying and differentially diagnosing pneumonia from other diseases. Improvements in biological markers and molecular testing techniques are becoming available. Despite shortcomings, biomarkers and molecular testing techniques have helped the early and differential diagnosis of infectious diseases like pneumonia. After diagnosing diabetic patients with community-acquired pneumonia using traditional techniques, biomarkers, and molecular strategies, certain valuable therapeutic approaches can be chosen which will reduce the mortality rates. Despite its shortcomings, this study sheds light on current and advanced methods for pneumonia identification and factors for acquiring and affecting the severity of pneumonia in diabetic patients. Indeed, randomized controlled trials (RCTs) can be used to assess the therapeutic role of biomarkers in future. Further, biomarkers can be utilized in the development of drugs.

## Figures and Tables

**Table 1:** Risk factors and their respective studies are summarized in Table 1. CAP: community-acquired pneumonia; DM: diabetes mellitus; GV: glycemic variability; SNP: single-nucleotide polymorphism; AGER: receptor for advanced glycaton endproducts; sRAGE: soluble receptor for advanced glycation endproducts; ARDS: acute respiratory distress syndrome; AECOPD: acute exacerbation of chronic obstructive pulmonary disease.

Risk Factor	Study	Total Subjects	Conclusion	Reference
Hyperglycemia	A retrospective cohort study	1318	An increase in blood glucose marks severity of CAP in patients without DM.	[40]
Glycemic variability	A retrospective study	282	Pneumonia patients in critical condition should have their early phase GV closely monitored.	[41]
Oxidative stress	A patient follow-up study	54	Damage to the redox state of erythrocytes and blood plasma components is crucial for the initiation and advancement of CAP processes.	[42]
Glycation and advanced glycation endproducts	Prospective study	500	In this cohort, AGER SNP rs2070600 (Ser/Ser) was linked to a greater plasma sRAGE and an elevated risk of ARDS.	[43]
Abnormal leucocyte	A retrospective case–control study	444	AECOPD was more likely to occur in patients with a monocyte proportion of >10% or <7.4% with an absolute count <0.62.	[44]
Impaired complement	A cross-sectional study	285	The severity of CAP patients is positively correlated with serum complement C3a at admission. Complement C3a is implicated in the pathophysiology of CAP, as evidenced by the attenuation of S. pneumoniae-induced inflammatory cytokine production in pulmonary epithelial cells with complement system inhibition.	[45]
Antibody response	A randomized, double-blind, placebo-controlled trial	270	Gimsilumab (a monoclonal antibody) did not reduce mortality or other important clinical outcomes in patients who had indications of systemic inflammation and COVID-19 pneumonia.	[46]
Micronutrient deficiency	A Nigerian tertiary health center	144	Incidence and severity of pneumonia in children from Nigeria were linked to low serum micronutrient levels, specifically zinc and vitamin A.	[47]
Metformin use	A retrospective analysis	22,638	Among Chinese people with diabetes, long-term metformin treatment was linked to a lower risk of pneumonia and pneumonia-related mortality.	[48]

**Table 2:** Important biomarkers involved in diagnosis of pneumonia.

Class of Biomarker	Name of Biomarker	Study Type and ID	Conclusion of Study	Reference
Inflammatory biomarker	Procalcitonin (PCT)	A prospective, international, study	Pneumonia diagnosis may be aided by procalcitonin, especially in patients with a high diagnostic ambiguity.	[167]
Inflammatory biomarker	CRP	Prospective hospital-based study	Pneumonia may use CRP as a clinical measure instead of TNF-alpha or IL-6.	[168]
Inflammatory biomarker	CRP	A prospective study	When treating pneumonia patients, serum CRP can help differentiate between endobronchial and parenchymal infections.	[169]
Inflammatory biomarker	Soluble triggering receptor expressed on myeloid cells-1 (sTREM-1)	A case–control study	In children who have undergone MV, serum sTREM-1 levels are a trustworthy biomarker for predicting VAP.	[170]
Inflammatory biomarker	Proadrenomedullin (proADM)	A prospective cohort study	Particularly in radiographic CAP, ProADM was linked to severe disease and distinguished between children who were reasonably well and those who did not acquire severe disease.	[171]
Inflammatory biomarker	MR-proANP (pro-atrial natriuretic peptide)	Prospective observational cohort study	MR-proANP levels are independent predictors of mortality in VAP and rise gradually with the severity of sepsis.	[172]
